# Supplementary material for: The Usefulness of Anthropometric Indices to Identify the Risk of Metabolic Syndrome
Source: Nutrients. 2019 Oct 29;11(11):2598. doi: 10.3390/nu11112598 (PMC6893758; doi:10.3390/nu11112598)
Supplement: Supplementary file 1 [file nutrients-11-02598-s001.pdf]

The Usefulness of Anthropometric Indices to Identify the Risk of Metabolic Syndrome

Table 1. Correlation coefficients between body size and shape.

Index	BMI r (p)	WHtR r (p)	%BF r (p)	ABSI r (p)	BRI r (p)	CUN-BAE r (p)
**TOTAL**						
Height	-0.037 (<0.001)	-0.055 (<0.001)	-0.382 (<0.001)	0.358 (<0.001)	-0.055 (<0.001)	-0.591 (<0.001)
Weight	0.818 (<0.001)	0.709 (<0.001)	0.303 (<0.001)	0.296 (<0.001)	0.709 (<0.001)	0.192 (<0.001)
WC	0.812 (<0.001)	0.916 (<0.001)	0.329 (<0.001)	0.612 (<0.001)	0.916 (<0.001)	0.228 (<0.001)
BMI	-					
WHtR	0.877 (<0.001)	-				
%BF	0.624 (<0.001)	0.519 (<0.001)	-			
ABSI	0.114 (<0.001)	0.498 (<0.001)	-0.160 (<0.001)	-		
BRI	0.877 (<0.001)	0.999 (<0.001)	0.621 (<0.001)	0.498 (<0.001)	-	
CUN-BAE	0.625 (<0.001)	0.492 (<0.001)	0.873 (<0.001)	-0.294 (<0.001)	0.492 (<0.001)	-
**MEN**						
Height	-0.066 (<0.001)	-0.225 (<0.001)	-0.087 (<0.001)	-0.002 (0.888)	-0.225 (<0.001)	-0.095 (<0.001)
Weight	0.858 (<0.001)	0.653 (<0.001)	0.673 (<0.001)	-0.002 (0.908)	0.653 (<0.001)	0.834 (<0.001)
WC	0.857 (<0.001)	0.931 (<0.001)	0.752 (<0.001)	0.438 (<0.001)	0.931 (<0.001)	0.856 (<0.001)
BMI	-					
WHtR	0.862 (<0.001)	-				
%BF	0.803 (<0.001)	0.767 (<0.001)	-			
ABSI	0.003 (<0.810)	0.431 (<0.001)	0.767 (<0.001)	-		
BRI	0.862 (<0.001)	0.999 (<0.001)	0.701 (<0.001)	0.431 (<0.001)	-	
CUN-BAE	0.991 (<0.001)	0.872 (<0.001)	0.808 (<0.001)	0.028 (0.075)	0.872 (<0.001)	-
**WOMEN**						
Height	-0.166 (<0.001)	-0.262 (<0.001)	0.050 (<0.001)	-0.020 (0.068)	-0.262 (<0.001)	-0.188 (<0.001)
Weight	0.905 (<0.001)	0.751 (<0.001)	0.860 (<0.001)	0.105 (<0.001)	0.751 (<0.001)	0.889 (<0.001)

WC	0.872 (<0.001)	0.964 (<0.001)	0.964 (<0.001)	0.534 (<0.001)	0.964 (<0.001)	0.870 (<0.001)
BMI	-					
WHtR	0.883 (<0.001)	-				
%BF	0.851 (<0.001)	0.770 (<0.001)	-			
ABSI	0.115 (<0.001)	0.517 (<0.001)	0.188 (<0.001)	-		
BRI	0.883 (<0.001)	0.999 (<0.001)	0.686 (<0.001)	0.517 (<0.001)	-	
CUN-BAE	0.993 (<0.001)	0.888 (<0.001)	0.851 (<0.001)	0.128 (<0.001)	0.888 (<0.001)	-

r - Spearman's rank correlation coefficients (p - value); BMI - Body Mass Index; WHtR - Waist-to-Height Ratio; %BF - Percent of Body Fat; ABSI - A Body Shape Index; BRI - Body Roundness Index; CUN-BAE - Clínica Universidad de Navarra-Body Adiposity Estimator.

Table 2. Unadjusted odds ratios and 95% confidence intervals for MetS.

**Indices**	Q	Men				Women			
		MetS classic (3 and more components from 5)		MetS modified (2 and more components from 4, other than WC)		MetS classic (3 and more components from 5)		MetS modified (2 and more components from 4, other than WC)	
		OR (95% CI)	p	OR (95% CI)	p	OR (95% CI)	p	OR (95% CI)	p
**BMI**	1(ref.)	1.0		1.0		1.0		1.0	
	2	3.08 (2.42-3.90)	<0.001	1.64 (1.35-2.00)	<0.001	3.04 (2.51-3.67)	<0.001	1.80 (1.53-2.10)	<0.001
	3	6.66 (5.26-8.42)	<0.001	2.59 (2.12-3.17)	<0.001	5.42 (4.51-6.52)	<0.001	2.76 (2.360-3.216)	<0.001
	4	11.15 (8.77-14.16)	<0.001	3.89 (3.17-4.78)	<0.001	8.78 (7.31-10.54)	<0.001	4.18 (3.58-4.87)	<0.001
	5	18.01 (14.03-23.11)	<0.001	5.99 (4.83-7.43)	<0.001	14.84 (12.33-17.85)	<0.001	7.06 (6.03-8.26)	<0.001
WHtR	1(ref.)	1.0		1.0		1.0		1.0	
	2	4.86 (3.76-6.29)	<0.001	1.89 (1.55-2.31)	<0.001	5.91 (4.70-7.45)	<0.001	2.112 (1.793-2.488)	<0.001
	3	9.52 (7.375-12.288)	<0.001	2.50 (2.05-3.05)	<0.001	11.37 (9.08-14.25)	<0.001	3.387 (2.886-3.974)	<0.001
	4	14.832 (11.45-19.22)	<0.001	3.62 (2.95-4.44)	<0.001	17.05 (13.61-21.36)	<0.001	5.064 (4.318-5.939)	<0.001

	5	26.04 (19.88-34.10)	<0.001	6.32 (5.08-7.85)	<0.001	31.54 (25.12-39.60)	<0.001	9.37 (7.96-11.02)	<0.001
%BF	1(ref.)	1.0		1.0		1.0		1.0	
	2	2.58 (2.05-3.25)	<0.001	1.64 (1.35-2.00)	<0.001	2.74 (2.28-3.30)	<0.001	1.90 (1.61-2.23)	<0.001
	3	5.41 (4.32-6.79)	<0.001	2.67 (2.18-3.26)	<0.001	4.80 (4.01-5.74)	<0.001	3.35 (2.86-3.92)	<0.001
	4	9.08 (7.22-11.43)	<0.001	3.82 (3.11-4.68)	<0.001	8.18 (6.84-9.78)	<0.001	4.82 (4.12-5.64)	<0.001
	5	12.63 (9.97-15.99)	<0.001	6.56 (5.27-8.16)	<0.001	12.22 (10.20-14.63)	<0.001	8.08 (6.88-9.48)	<0.001
ABSI	1(ref.)	1.0		1.0		1.0		1.0	
	2	1.59 (1.31-1.94)	<0.001	1.13 (0.93-1.37)	0.235	1.82 (1.55-2.13)	<0.001	1.51 (1.31-1.75)	<0.001
	3	1.95 (1.60-2.38)	<0.001	1.23 (1.01-1.49)	<0.001	2.57 (2.20-3.00)	<0.001	2.03 (1.751-2.344)	<0.001
	4	2.15 (1.77-2.62)	<0.001	1.34 (1.098-1.623)	0.004	3.34 (2.87-3.90)	<0.001	2.465 (2.13-2.85)	<0.001
	5	2.79 (2.29-3.41)	<0.001	1.56 (1.28-1.90)	<0.001	4.43 (3.80-5.16)	<0.001	3.17 (2.74-3.67)	<0.001
BRI	1(ref.)	1.00		1.0		1.0		1.0	
	2	2.57 (2.06-3.22)	<0.001	1.58 (1.30-1.93)	<0.001	3.99 (3.25-4.90)	<0.001	2.02 (1.71-2.37)	<0.001
	3	4.55 (3.651-5.673)	<0.001	2.21 (1.81-2.69)	<0.001	7.42 (6.07-9.06)	<0.001	3.26 (2.78-3.82)	<0.001
	4	6.992 (5.59-8.74)	<0.001	2.97 (2.43-3.63)	<0.001	11.66 (9.55-14.23)	<0.001	4.81 (4.11-5.64)	<0.001
	5	12.28 (9.72-15.51)	<0.001	4.84 (3.92-5.99)	<0.001	21.30 (17.40-26.06)	<0.001	8.77 (7.46-10.31)	<0.001
CUN-BAE	1(ref.)	1.0		1.0		1.0		1.0	
	2	2.91 (2.30-3.70)	<0.001	1.73 (1.42-2.11)	<0.001	3.25 (2.66-3.96)	<0.001	1.85 (1.58-2.17)	<0.001
	3	6.52 (5.16-8.23)	<0.001	2.72	<0.001	6.84	<0.001	2.71 (2.32-3.17)	<0.001

				(2.22-3.32)		(5.65-8.29)			
	4	10.62 (8.37-13.48)	<0.001	3.89 (3.17-4.78)	<0.001	10.49 (8.66-12.70)	<0.001	4.32 (3.70-5.05)	<0.001
	5	19.04 (14.82-24.47)	<0.001	5.39 (4.36-6.67)	<0.001	17.62 (14.52-21.38)	<0.001	6.41 (5.48-7.49)	<0.001

OR – odds ratio; CI – confidence interval; Q – quintile; WC – waist circumference; ref. – reference level; BMI - Body Mass Index; WHtR - Waist-to-Height Ratio; %BF - Percent of Body Fat; ABSI - A Body Shape Index; BRI - Body Roundness Index; CUN-BAE - Clínica Universidad de Navarra-Body Adiposity Estimator.
